# Sexual Dysfunction in Alopecia Areata: A Systematic Review

**DOI:** 10.3390/jcm14082602

**Published:** 2025-04-10

**Authors:** Piotr K. Krajewski, Aleksandra Złotowska, Jacek C. Szepietowski, David Saceda Corralo

**Affiliations:** 1 University Centre of General Dermatology and Oncodermatology, Wroclaw Medical University, 50-556 Wroclaw, Poland; aleksandra.zlotowska@student.umw.edu.pl; 2 Department of Dermatovenereology, 4th Military Hospital, 50-981 Wroclaw, Poland; jacek.szepietowski@pwr.edu.pl; 3 Faculty of Medicine, Wroclaw University of Science and Technology, 50-377 Wroclaw, Poland; 4 Servicio de Dermatología, Hospital Universitario Ramón y Cajal, IRyCIS, 28034 Madrid, Spain; d.saceda@gmail.com; 5 Hair Disorders Unit, Grupo Pedro Jaén, 28002 Madrid, Spain

**Keywords:** alopecia areata, sexual health, sexual disorders, sexual dysfunction

## Abstract

**Background:** Alopecia areata (AA) contributes to clinically significant suffering, and impaired social functioning. Among AA patients, there is a clear impact of the disease on their sense of attractiveness and desirability as sexual partners. This review explores the development of sexual disorders among AA patients, highlighting their importance in the clinical diagnosis of comorbid health disorders with hair loss. **Methods:** A systematic review was conducted by searching electronic databases, including MEDLINE and Google Scholar, without date limitations, according to the PRISMA guidelines. Key search terms included “sexuality” or “sexual health” or “sexual dysfunction” or “sexual disorder” AND “alopecia areata”. Data synthesis included findings from eight relevant studies. **Results:** Hair loss in the course of AA has a negative impact on the sexual sphere, significantly reducing the quality of life of patients and their partners. Proper sexual functioning is an integral part of every person, so special attention should be paid to the possibility of developing sexual dysfunction in the course of AA. **Conclusions:** Small sample sizes and heterogeneous populations make it difficult to draw firm conclusions. Continued research with standardized criteria for SD diagnosis and appropriately large cohorts will be essential to fully establish psychosexual disorders among AA patients.

## 1. Introduction

Alopecia areata (AA) is a chronic and debilitating inflammatory disease that affects approximately 2% of the population [1,2]. It can affect all age groups, with a peak incidence between 25 and 37 years of age [3]. Clinically, AA is characterized by localized patches of hair loss on the scalp [1]. AA is marked by a progressive course leading to complete hair loss on the scalp and body, significantly impacting patients’ quality of life [4]. Autoimmune reactions to anagen hair follicles (HFs) play a key role in the pathogenesis [5].

AA remains challenging to treat because of its chronic, relapsing nature. Current treatments focus on halting disease progression, promoting regrowth, and maintaining hair growth [3]. Despite the availability of various treatment methods, many patients experience suboptimal outcomes due to what some studies describe as a limited impact on the disease course [6].

For many people, hair is an important element in shaping self-identity, building self-confidence, and self-esteem [7]. It is inextricably linked to self-image and the way one is perceived by others [8]. Hair loss contributes to clinically significant suffering, impaired social and professional functioning, numerous mental disorders, and a negative impact on HRQoL [9,10]. Consequently, many patients experience harassment, stigmatization, and social discrimination [9]. Low self-esteem and frustration associated with AA lead to frequent co-occurrence of depression, generalized anxiety disorder, social phobia, post-traumatic stress disorder, suicidal ideation, and psychosexual disorders [11].

Sexual health is defined by the World Health Organization as “a state of physical, emotional, mental, and social well-being in relation to sexuality; it is not merely the absence of disease, dysfunction, or infirmity. Sexual health requires a positive and respectful approach to sexuality and sexual relationships, as well as the possibility of having pleasurable and safe sexual experiences, free of coercion, discrimination and violence” [12]. Clinically significant disorders of sexual behavior and sexual pleasure fall under sexual dysfunction (SD), a heterogeneous group of disorders involving deviations from normal sexual functioning [13]. A special form of SD is psychosexual disorders, attributed to mental problems without a discernible pathological physical cause. They can result from guilt, stress, anxiety, nervousness, worry, fear, depression, distorted body image, or physical/emotional trauma. The clinical picture varies depending on gender and individual factors [14].

Hair loss in AA often triggers a negative perception of one’s own body—a sense of unattractiveness and shame—that correlates with the development of psychosexual disorders [15,16]. AA patients may experience decreased sexual desire, arousal, sexual satisfaction, and erectile dysfunction, the severity of which depends on the stage of the disease and the importance of hair in shaping the individual’s identity [15].

Given the importance of sexual health, we conducted a systematic review exploring the development of sexual disorders among AA patients, highlighting their relevance in the clinical diagnosis of comorbid health disorders with hair loss.

## 2. Methodology and Search Strategy

**Figure 1:**
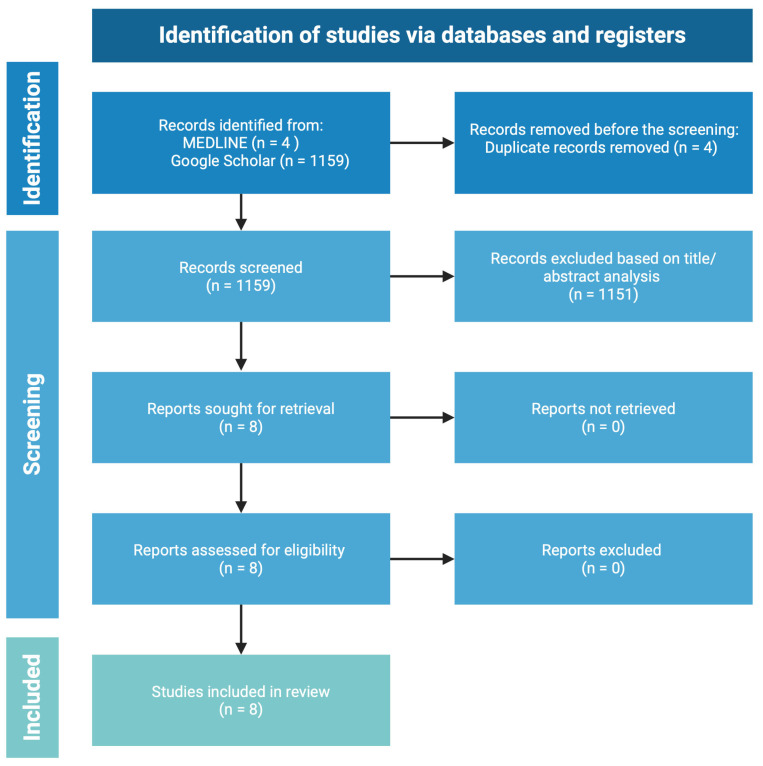
Literature search carried out according to the PRISMA guidelines.

This systematic review was performed following the PRISMA guidelines [17]. The MEDLINE and Google Scholar databases were systematically searched in December 2024 without any additional filters. The main keywords used were “sexuality” or “sexual health” or “sexual dysfunction” or “sexual disorder” AND “alopecia areata”. All of the papers identified by keywords were reviewed, and additional relevant articles were purchased for inclusion in the study with no restrictions of patient age, sex, ethnicity. Only comparative studies were included. The list of referenced downloaded publications was searched for additional information about the sources. The exclusion criteria were as follows: full text not available, full text not in English, studies with duplicate information published elsewhere, and non-original data such as reviews, commentaries, or editorials. Studies considered ineligible included articles that did not contain new data and summarized previously published data. This systematic review acknowledges the potential for bias in the selection and interpretation of studies. Potential sources of bias in the identified studies are acknowledged including the small size of patient cohorts and heterogeneous populations. Efforts were made to minimize it through a comprehensive search strategy, predefined inclusion criteria, and critical appraisal of the included studies. Two independent authors conducted the search (PK and AZ). In total, 8 articles were included in the final review. The search strategy is presented in Figure 1. Ethics approval was not required for this study.

## 3. Alopecia Areata (AA)

AA is a complex, multifactorial disease of autoimmune origin. It is the second most common type of non-scarring alopecia, after androgenetic alopecia (AGA) [18]. The course of the disease is variable and unpredictable, with periods of relapses and remissions, ranging from mild to severe or diffuse forms [19]. Its etiopathogenesis involves intricate interactions between genetic predispositions, environmental factors, and immune system dysregulation [20,21]. Key features include immune privilege collapse, activated interferon-γ (IFN-γ) signaling pathways, and activated pathways of cytotoxic CD8+ T lymphocytes [21].

Symptoms depend on the disease stage and individual predispositions. The clinical picture typically manifests as patchy hair loss or diffuse involvement of hair on the scalp or the entire body [22]. In the most common form of AA, one or more well-defined round or oval patches of hair loss on the scalp predominate. Characteristic smaller 3 to 4 mm “exclamation mark” hairs may be seen at the periphery of the patch [23]. In 30–50% of cases, the patchy form of AA remits spontaneously within 6–12 months. By contrast, the spontaneous remission rate for alopecia totalis is <10%. Patients have a 100% risk of relapse within 20 years, regardless of the disease form [24].

The prevalence of AA in the general population is 1–2% [25]. AA can appear at any age and affects both genders and all ethnicities [22]. The lifetime risk of disease onset is estimated at 1.7–2.1%. In most cases, AA affects adults, although it can occur in all age groups [25,26]. Women are more likely to develop the disease, with a noticeable prevalence in those whose disease begins after the age of 50 [2]. Approximately 60% of patients notice the first small, well-defined patch of hair loss on the scalp or beard before the age of 30 [26,27].

### 3.1. Sexual Health

Sexual health is a complex, dynamic process shaped by neurological, endocrine, and vascular systems [28]. Sexuality, intimacy, and sexual identity are integral spheres for the proper development of personality, communication, and love in every person [29]. Clinical aspects related to sexual health include sexual satisfaction, sexual self-efficacy, sexual self-esteem, and sexual pleasure [29,30]. Disruptions in any of these phases of sexual response can lead to sexual dysfunctions, affecting both physical and mental health [14,31].

Impaired sexual functions of psychological origin are referred to as psychosexual disorders. Their development is influenced by mental disorders, socio-cultural factors, and physical or emotional trauma. Symptoms vary depending on the individual and gender [14,32,33]. In men, erectile dysfunction, ejaculatory dysfunction, and hypoactive sexual desire disorders are common. Women may experience genital pain syndromes, vulvodynia, vaginismus, and hypoactive sexual desire disorders [14,34,35].

Psychosexual disorders often co-occur with chronic physical and mental health conditions [14,36,37,38]. Adverse effects of treatments for these conditions can also negatively impact sexual health [14,37,38]. A study by Field et al. [39], involving 15,162 participants aged 16–74, found a correlation between mental/physical health disorders, reduced sexual activity, and reduced sexual satisfaction at all ages [39]. Similarly, Flynn et al. [40], in a cross-sectional questionnaire study of 3515 adults, showed lower sexual satisfaction among individuals with health disorders. They emphasized the importance of sexual health and satisfaction with sexual life as a crucial aspect of overall quality of life [40].

Emerging evidence suggests that dermatoses independently increase the risk of psychological disorders due to the visible skin lesions and chronic nature of these conditions. About 23.1% of patients with dermatoses develop impairments in sexual life [41]. This is observed in chronic dermatological conditions like psoriasis, atopic dermatitis, hand eczema, and AA. The unsightly effect of such lesions leads to low self-esteem, shame, stigmatization, and reduced confidence, potentially resulting in sexual dysfunction [14]. In a systematic review and meta-synthesis by Barisone et al. [42], visible dermatological lesions induced negative emotions in both men and women, driven by fear of external judgment. A deep sense of shame and social stigmatization contributed to suicidal ideation in some patients. The authors highlighted the significant impact of these conditions on perceived unattractiveness, fostering avoidance of sexual intimacy and development of psychosexual disorders [42].

To objectively assess the existence or severity of SD, general dermatological quality-of-life indicators such as the Dermatology Life Quality Index (DLQI) (question 9: “Over the last week, how much has your skin caused any sexual difficulties?”) or Skindex (question 29: “My skin condition interferes with my sex life”) are sometimes used. Their drawback is that they do not differentiate between various forms of sexual dysfunction. Identifying sexual disorders is crucial for improving patients’ quality of life, so specialized tools have been developed to assess sexual functioning more accurately [43]. These tools are objective, standardized, accurate, and reliable. Proper training is key to interpreting their results correctly [44]. Commonly used screening instruments in clinical practice for female sexual disorders include the Sexual Quality of Life-Female (SQoL-F), the Female Sexual Function Index (FSFI), and the Female Sexual Distress Scale (FSDS). For males, the Sexual Quality of Life-Male (SQoL-M), the International Index of Erectile Function (IIEF), and the Male Sexual Health Questionnaire (MSHQ) are employed [44,45,46,47,48,49,50]. Because these tools focus on different aspects of psychosexual disorders, comparing study outcomes that use different methods remains challenging.

### 3.2. The Association Between Sexual and Mental Health

Significant emotional suffering, impaired individual functioning, and the development of mental disorders can escalate following hair loss. AA patients are at a higher risk of developing psychiatric disorders than the general population. Mental disorders can, in turn, cause disturbances in various areas of sexual life, such as desire, arousal, or sexual satisfaction [51,52].

In a systematic review by Herder et al. [53] involving 24 studies, the estimated prevalence of sexual dysfunction in psychiatric disorders ranged from 45% to 93% for depressive disorders, 33% to 75% for anxiety disorders, 25% to 81% for obsessive–compulsive disorders, and around 25% for schizophrenia. The authors noted that these findings were limited by small sample sizes and the use of multiple (sometimes unvalidated) questionnaires, possibly affecting the results’ validity [53]. Sexual dysfunction can also be a side effect of antidepressant treatment or arise from nonadherence to medical recommendations [54]. Epidemiological and clinical studies show that depression is associated with impaired sexual function and satisfaction, even in patients not receiving treatment. The prevalence of reduced libido, erectile issues, or vaginal dryness correlates with the severity of the psychiatric disorder [55].

A study by Field et al. [56] involving 1331 patients aged 16–64 found that depression was strongly associated with reduced sexual function and increased use of sexual health services [56]. Meanwhile, patients with anxiety disorders often experience greater difficulties with intimacy and fear of rejection, leading to avoidance of intimacy and withdrawal from relationships [57]. Carr et al. [58] observed, among young men who have sex with men, that depression and stress correlated with worsened sexual functioning, lower sexual satisfaction, and increased anal discomfort [58]. A study by Bodenmann et al. [59] found that chronic stress negatively correlates with sexual activity, satisfaction, fulfillment, and relationship quality [59]. Likewise, in AA patients, mental health disorders and severe psychosocial stress can impair sexual function [60].

### 3.3. Sexuality of AA Patients

The effects of AA are distressing for both men and women, negatively influencing social functioning, emotional well-being, and self-esteem [61].

Hair loss is linked to diminished self-confidence, lower self-esteem, and a poorer body image, particularly among women [61,62]. In men, AA significantly affects the sense of attractiveness and desirability as a sexual partner, with particularly pronounced effects seen in younger men [62]. Li et al. [63] conducted a survey using the online version of validated SQOL-F and SQOL-M questionnaires in 64 women and 17 men with AA. They noted a marked decrease in sexual quality of life in both men and women. The mean SQOL-F score was 51.3 ± 22.9, whereas the mean SQOL-M score was 62.7 ± 33.9. This difference was not statistically significant. AA had a significant impact on embarrassment among women and anxiety among men. Regardless of gender, many respondents strongly identified with the statement “I feel like I have lost something” [63].

A cross-sectional study by Barba et al. [64], involving 60 patients with AA and 60 healthy controls, showed that female AA patients had a higher sexual dysfunction (SD) index than male AA patients and healthy controls. The high incidence of SD in female AA patients correlated with younger age, shorter disease duration, and higher rates of anxiety and depression. In men, SD more commonly co-occurred with older age and more severe AA [64]. However, a study by Diaz et al. [65] with 60 patients and 60 controls found no effect of AA on IIEF-5 and FSFI-6 scores. The mean IIEF-5 score was 21 ± 1.01, and the mean FSFI-6 score was 13.95 ± 1.42. In the control group, the IIEF-5 and FSFI-6 were slightly higher, with no significant difference between the groups. Nonetheless, the study noted that SD in female AA patients occurred more often than in healthy women, which was not observed in men [65].

AA also negatively affects romantic relationships, often leading to the breakdown of existing partnerships or to avoidance of forming close bonds if acceptance is uncertain [66]. The impact on partners’ sexual quality of life depends on the extent of hair loss and the severity of a patient’s emotional distress [67,68]. Several studies have shown a significant association between AA and relationship difficulties. In a Spanish cross-sectional study by Diaz et al. [67], 42 AA patients with varying disease severity and their 42 cohabitants completed the IIEF-5, FSFI-6, and a numeric rating scale (NRS) for sexual impairment. Results reveal that 69% of AA patients had impaired sexual function, compared to 57.1% of cohabitants. Poor quality of life, longer disease duration, and higher anxiety and depression in AA patients negatively influenced their partners’ assessment of sexual life. Additionally, frustration with disease relapses caused patients and their partners to underestimate their sexual lives [67].

Another study by Aldhouse et al. [69], involving 45 patients aged 15–72, all with ≥50% scalp hair loss as per the SALT score, found both negative and positive effects of AA on relationships with partners, family, or friends. Some patients avoided intimate relationships due to fear of non-acceptance and low self-esteem. Others hid the diagnosis from romantic partners to suppress feelings of embarrassment or rejection. AA did not seem to disrupt family dynamics—family members often showed support. However, some individuals withdrew from social life and severed friendships due to fear of judgment [69].

A 2023 scoping review by Muntyanu et al. [70] also found that AA complicates the formation and maintenance of romantic relationships. Hair loss made patients feel less attractive, significantly lowering sexual quality of life regardless of marital status. The fear of rejection led many to hide their diagnosis, sometimes resulting in relationships ending. Feeling helpless, having low self-esteem, and reduced self-confidence often drove patients to camouflage their baldness—even with their partners. This negative self-image and avoidance of social contact exacerbated their reduced quality of life and worsening mental health [70].

The importance of sex life as a key component of mental health is supported by the correlation between sexual satisfaction and emotional stability [65]. Kim et al. [71] conducted a retrospective study of 7706 adult patients from 2002 to 2013, investigating how AA influenced the development of psychiatric disorders (PD). Results indicate that AA patients were not significantly more likely to develop nonorganic SD compared to controls. The authors emphasized that the severity and duration of AA might play a role in the development of psychiatric disorders, including SD. Moreover, AA and PD interacted by intensifying each other’s severity [71].

Hair also plays a key role in men’s self-image. Beards and facial hair can be associated with social dominance, aggressiveness, and attractiveness—major attributes of masculinity [72]. Loss of facial and body hair negatively affects men’s self-esteem and confidence [7]. Sexual orientation can also influence concern about appearance; homosexual men often report greater sensitivity to negative judgments about appearance than heterosexual men, emphasizing the role of physical attractiveness in attracting partners [73].

A study by Zucchellii et al. [74] of 177 men (aged 17–79), including 83 with AGA and 94 with AA, assessed the psychosocial burden of hair loss in men. Despite distinct patterns of hair loss in AGA vs. AA, both groups showed similarly increased anxiety, lower happiness, reduced sense of purpose, and lower life satisfaction. Patients frequently reported discrimination, stigmatization, a damaged self-perception of attractiveness, social isolation, and relationship problems. Further analysis shows that homosexual, bisexual, or other sexual minority men felt more appearance-related pressure than heterosexual men and were more distressed by hair loss. Lack of hair was perceived as making them less desirable in the gay community. Consequently, these sexual minorities may be particularly vulnerable to AA’s psychosocial impact, underscoring the need for further research [74].

**Figure 2:**
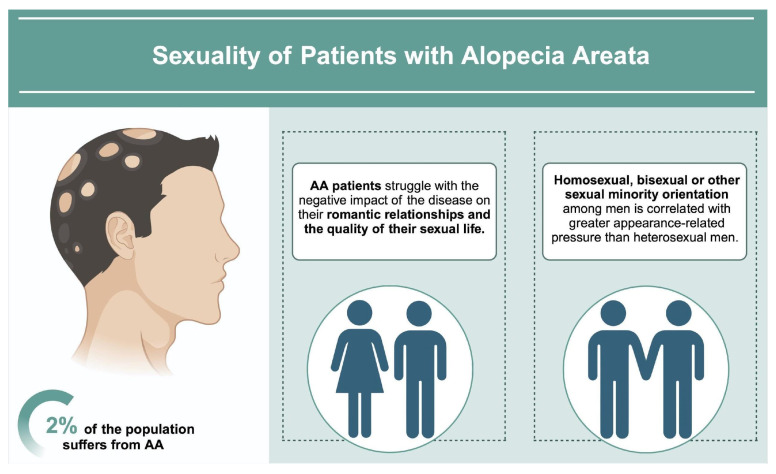
The impact of alopecia areata on patients’ romantic relationships and sexuality.

The results are summarized in Table 1. The impact of AA on patients’ romantic relationships and sexuality is presented in Figure 2.

### 3.4. Impact of Alopecia Areata Treatment on Sexual Health

Therapeutic strategies often fail to provide sustained disease control in cases of rapid hair loss, more extensive AA (≥50% hair loss), chronic disease, or a combination thereof [18,75,76]. Currently available treatments for extensive AA neither offer consistently long-term effectiveness nor a favorable side-effect profile. Many patients discontinue therapy due to dissatisfaction with results [18,77]. The visibility of hair loss, high relapse rate, and limited treatment options likely contribute to a higher prevalence of psychosexual disorders in AA patients than in the general population [64].

Sexual dysfunction in AA may also arise from negative effects of pharmacological treatment. Methotrexate—an immunosuppressive antifolate agent commonly used in AA—can produce gastrointestinal disturbances, malaise, and headaches [78,79]. It may also affect spermatogenesis, reduce libido, and contribute to infertility [80]. Theodosiou et al. [81] reported two psoriasis cases where 12.5 mg methotrexate plus folic acid was associated with reduced libido, difficulty achieving erections, and erectile dysfunction, all of which resolved upon discontinuation [82]. Although the precise mechanism is unclear, methotrexate-induced inhibition of interleukin-1 (IL-1) may interfere with proper pituitary function, while nitric oxide inhibition hampers smooth muscle activity [80,81]. Supplementing folic acid lessens many side effects, but its role in preventing sexual dysfunction is unexamined [82].

Cyclosporine, a calcineurin inhibitor widely used in autoimmune diseases, has similarly been implicated in erectile dysfunction [83]. In a cross-sectional survey by Tial et al. [84] on male renal transplant recipients, cyclosporine use was linked with higher rates of erectile dysfunction compared to other immunosuppressive regimens [84]. Systemic corticosteroids have also been implicated in sexual dysfunction [82]. Dexamethasone at 1 mg/day led to impotence in a 34-year-old patient with congenital adrenal hyperplasia, which resolved when the dose was lowered to 0.5 mg/day [85]. An observational study in 17 men treated with 10 mg/day methylprednisolone found erectile dysfunction in 58% and decreased libido in 52% [86].

Nonetheless, some medications used to treat AA can benefit sexual health. Dupilumab, a monoclonal antibody against IL-4 and IL-13 that inhibits Th2 lymphocyte signaling, has shown promise in treating AA [87]. In a prospective study of 170 atopic dermatitis patients (51.8% men; mean age 33.78 ± 12.21 years), dupilumab significantly improved sexual desire as measured by the Sexual Desire Inventory-2 (increasing from 33.57 ± 8.18 to 56.34 ± 16.5). Differences between genders were minor and not statistically significant [88]. Statins, which inhibit IFN-γ signaling and lymphocyte activation, also show favorable results in acute AA—particularly the simvastatin/ezetimibe combination [89]. A meta-analysis of 586 men over 40 demonstrated that statins could ameliorate erectile dysfunction by lowering lipid levels, with a mean IIEF improvement of 3.27 points (*p* < 0.01) [90].

Providing comprehensive and holistic care for AA patients is a key component of therapeutic treatment [91]. Psychological interventions such as mindfulness, hypnosis, cognitive behavioral therapy and psychotherapy are applicable to patients refractory to pharmacological treatment. Findings indicate that they improve mental health, quality of life indicators and, consequently, also affect hair regrowth. They also aim to provide stabilization of clinical status in terms of sexual disorders, depression, anxiety, and self-confidence [92,93]. In addition, cognitive behavioral therapy helps to change maladaptive negative beliefs and to acquire coping skills to deal with negative emotional states and difficult situations [91].

## 4. Study Limitations

Most studies suggest a statistically significant association between AA and sexual disorders; however, small sample sizes and heterogeneous populations limit definitive conclusions. In addition, of the studies reviewed, only one was concerned with a large community of patients. Reasons for heterogeneity included differences in the study populations (age, gender, health status), study biases (different measurement methods, study duration), and different rules for classifying results. Further research with standardized criteria for diagnosing SD and larger cohorts is necessary to fully delineate psychosexual disorders among AA patients.

## 5. Conclusions

Sexuality is a natural bodily function and one of the main motivations for forming connections and bonds. A sense of value, dignity, and self-confidence in one’s role as a partner relies on both the psychological desire for sex and the physical ability to engage in sexual activity [94,95]. Hair loss in AA negatively affects the sexual domain, significantly reducing the quality of life of both patients and their partners.

Because sexual functioning is an integral part of overall well-being, clinicians should remain vigilant for the potential development of sexual dysfunction in AA. Collaboration among all specialists involved in AA treatment should begin as early as possible, to facilitate timely detection and management of coexisting disorders. A multidisciplinary team—comprising dermatologists, psychologists, psychiatrists, and sexologists—may be particularly effective in preventing heightened discomfort in intimate relationships and reducing avoidance of sexual contact.

## Figures and Tables

**Table 1:** Summary of research results on AA patients’ sexuality.

Author (Year)	Study Population (AA Patients)	Mean Age	SD Measurement Method	Results
Li et al. [63] (2018)	81 (60)	39.7 ± 13.8 years for women 37.4 ± 9.9 years for men	SQOL-F and SQOL-M	The study found a significant decrease in the quality of sexual life in both women and men. The mean SQOL-F score was 51.3 ± 22.9, while the mean SQOL-M score was 62.7 ± 33.9. The difference was not statistically significant between these two cohorts (*p* = 0.12). AA was found to have a significant impact on the feeling of embarrassment in women (n = 48, 75.0%) and on anxiety in men (n = 7, 46.7%).
Barba et al. [64] (2024)	120 (60)	*	Numerical scale and gender-specific questionnaires	The study reveals a higher SD index in female AA patients than in male AA patients and healthy individuals (*p* < 0.005). The high incidence of SD in female AA patients correlated with younger age, shorter duration of illness, and higher rates of anxiety and depression (*p* < 0.05). In contrast, in males, sexual dysfunction usually co-occurred with older age and greater severity of AA (*p* < 0.05).
Diaz et al. [65] (2022)	120 (60)	AA patients: 39.68 ± 13.15 Controls: 40.21 ± 12.79	IIEF-5 and FSFI-6	The mean IIEF-5 score was 21 ± 1.01, and the FSFI-6 score was 13.95 ± 1.42. In the control group, the IIEF-5 and FSFI-6 scores were slightly higher, 21.27 ± 0.92 and 16.35 ± 1.47, respectively. The differences between the groups were not statistically significant (*p* > 0.05). Results reveal that SD in female AA patients (66.7% female patients) occurs more often than in healthy individuals (42.2% female controls), but this was not observed in the opposite sex (SD 33.3% male AA patients, 40% male controls).
Diaz et al. [67] (2022)	84 (42)	AA patients: 40.59 ± 8.92 Cohabitants: 46.76 ± 12.10	IIEF-5, FSFI-6, and numeric rating scale (NRS) for sexual impairment	The results show impaired sexual function in 69% of the patients (29/42): 78.8% (26/33) females and 33.3% (3/9) males and in 57.1% of the cohabitants (24/42): 60% (9/15) females and 50% (15/30) males. Low quality of life, longer duration of the disease, higher anxiety and depression scores in AA patients negatively influenced the assessment of sexual life of their partners (*p* < 0.05).
Aldhouse et al. [69] (2020)	45 (45)	33.3 years	A semi-structured interview guide, developed with expert clinician input, including open-ended questions to explore patients’ experiences of living with AA	Some patients admitted to avoiding entering into closer relationships due to fear of lack of unconditional acceptance and low self-esteem due to AA. In addition, patients described hiding the disease from romantic partners due to suppressing fear of embarrassment and rejection.
Kim et al. [71] (2020)	38,530 (7706)	Unknown	Psychiatric disorder codes F01–F99 and all their subclassification codes	AA patients were not more likely to develop nonorganic SD compared to healthy individuals (HR healthy patients—1; HR AA patients—2.01; *p* = 0.569).
Zucchelli et al. [74] (2024)	177 (94)	47.05	Cross-sectional online survey with closed and open-ended questions	Homosexual, bisexual, or other sexual minority orientation among men correlated with greater appearance-related pressure than heterosexual men and with increased complexes due to hair loss.

* Only abstract available. SD = sexual disorders.

## Data Availability

No new data were created or analyzed in this study.

## Abbreviations

AA	Alopecia areata
HRQoL	Health-related quality of life
SD	Sexual dysfunction
IFN-γ	Interferon-γ
SQol-F	Sexual Quality of Life-Female
FSFI	Female Sexual Function Index
FSDS	Female Sexual Distress Scale (FSDS).
SQoL-M	Sexual Quality of Life-Male
IIEF	International Index of Erectile Function
MSHQ	Male Sexual Health Questionnaire
PD	Psychiatric disorders
Il-1	Interleukin 1
Il-4	Interleukin 4
Il-13	Interleukin 13
ED	Erectile dysfunction
AGA	Androgenetic alopecia
